# 

**Published:** 1821-12

**Authors:** 


THE LONDON
### Medical and Physical Journal.

6 OF VOL. XLVI.] DECEMBER, 1821. [NO. 274.

NOTICE.

*Another of the series of* PRÖEMIA *to the several volumes of this Journal, (which commenced with that to the forty-third volume,) comprising a History of the Progress of Medicine and its auxiliary Sciences for the half-year immediately previous to the period of their production, respectively, was published on the last day of July. One of the especial intentions of those* Pröemia, *is to present a comprehensive view of the state and progress of Medicine throughout Europe generally, and in the United States of America; an object that cannot be effected in the regular monthly Numbers of the Journal, because of the small extent of space which can be there appropriated to this purpose.*

### MONTHLY CATALOGUE OF MEDICAL BOOKS.

A Treatise on the Diseases of the Chest. Translated from the French of R. T. H. Laennec, M.D. with a Preface and Notes, by John Forbes, M.D. 1 vol. 8vo. 14s.

Reflections on Gall and Spurzheim's System of Physiognomy and Phrenology. By John Abernethy, F.R.S. 8vo. 3s.

A System of Pathological and Operative Surgery, founded on Anatomy. By Robert Allan, F.R.S. &c. Vol. II. 12s. 6d.

The Accoucheur's Vade-Mecum. By J. Hopkins, M.D. 2 vols. 12mo. 10s. 6d.

The London Practice of Midwifery. 12mo. 6s. boards.

Popular Compendium of Anatomy. By Wm. Burke. With Plates. 6s.

A Chemical Chart, or Table. By Robert Crowe, M.D. Sheet folio, 5s. 6d.

[HIGHLEY, FLEET-STREET.]

### NOTICES TO CORRESPONDENTS.

Dr. G. GREGORY *has addressed a letter to us, in which he states that, in the opinion of his friends, a particular passage which appeared in our recent review of his three papers in the* Medico-Chirurgical Transactions, *"reflects equally upon the character of the Society and of himself personally."* Dr. GREGORY *ought to know us better. In our Reviews we never meddle with the* personal *character of authors. Their* characters as writers *they themselves commit to the keeping of their readers; and is it singular that it should be occasionally investigated? Does not the Doctor himself write reviews? aye, and very clever ones too; and is he always gentle with his author? "Thou criest mercy, when thou shewest none." As for the character of the Society, we challenge* Dr. G. GREGORY *to point out a single critical publication other than our own, in which their character as a body has been better upheld, or more justly appreciated.*

Dr. DE RENZY, *of Dublin, will perceive that we have complied with his request.*

*The communication from Hampton-Court Barracks is not of a nature to be admitted in our Journal.*

*We will take an early opportunity of attending to the suggestion of* Dr. GEORGE EDWARD MALE.

Mr. TWEEDALE, *and* Mr. ORTON *of Madras, have our best thanks for their respective communications.*

*We have lately been pestered with solicitations to take notice of, and expose to the world, the tricks by which certain individuals, with purchased diplomas, contrive to evade the restrictive laws of the College, and practise as regular physicians in the very teeth of the censors. We must, however, decline the honour of meddling with this tribe; and can only join our Correspondents in repeating the prayer which* Othello *pronounced on a different occasion:*

> "Oh, heavens! that such *empiricks* thou'dst unfold,
> And put in every honest hand a whip,
> To lash the rascals naked through the world."

*Although voluminous, we have contrived to insert the documents transmitted to us by* Dr. MAYO, *on the subject of the Winchester Eye Infirmary. This we have done, because we deem it necessary, as journalists, to maintain the strictest impartiality. At the same time, it is for the contending parties to be discreet in their demands on the Editors of Journals, and to have some pity on the readers of those publications.*

*We have been requested by* Mr. W. WRIGHT, *of Henrietta street, Covent Garden, to announce to our readers that the first Number of a quarterly publication, containing the Transactions of a new Medical Society, called the Society of Practical Medicine of London, will be published on the 1st of January,* 1822.

*Though transmitted to us very late in the month,* Dr. SCUDAMORE'S *reply to* Mr. BAMPFIELD *has been inserted. This will show our anxiety to have the subject of controversy between these two gentlemen fairly investigated. The question itself is one of great importance, and by no means settled, whatever may be the individual opinion of the two writers. We are confident that the discussion will be carried on with perfect decorum, and with those dignified feelings of reciprocal respect which gentlemen, professing the same liberal art, should ever entertain for each other.*

A REFORMER *has some secret object in view, in desiring us to insert his communication; which we beg leave to decline. The characteristic humour of the facetious writer, whose style he has attempted to imitate, is far above the reach of* REFORMER'S *genius. In his communication, vulgar scurrility stands as the sorry substitute of wit; and personal abuse usurps the place of satire.*

T. O. S. *must have written his letter after dinner. On perusing it, we could not help reading his initials backwards.*

*A Correspondent from the* NORTH, *who writes in a disguised hand, and in terms strongly and personally hostile to us, loudly condemns the recent change in the Editorship of this Journal. In answer, we have only to observe, that, by increasing exertions in conducting the Journal to the satisfaction of the public, we hope to be able to give the lie to his* prognostic *of our inevitable ruin. By the bye, what an egregious dunce our Correspondent must be, to get a frank for his letter from the gentleman at whose house he is known to be a frequent visitor! This trick smells more of the south than of the* NORTH.

*In our January Number we shall have the honour of submitting to our Readers some few improvements in the arrangement of the contents of this Journal.*

